# Color restoration based on digital pathology image

**DOI:** 10.1371/journal.pone.0287704

**Published:** 2023-06-28

**Authors:** Guoxin Sun, Xiong Yan, Huizhe Wang, Fei Li, Rui Yang, Jing Xu, Xin Liu, Xiaomao Li, Xiao Zou

**Affiliations:** 1 School of Clinical Medicine, Qingdao University, Qingdao, China; 2 Department of Pathology, Qingdao Central Hospital, Qingdao, China; 3 School of Computer Engineering and Science Shanghai University, Shanghai, China; 4 Department of Breast Surgery, Xiangdong Hospital Affiliated to Hunan Normal University, Hunan, China; Universidad de Guadalajara, MEXICO

## Abstract

**Objective:**

Protective color restoration of faded digital pathology images based on color transfer algorithm.

**Methods:**

Twenty fresh tissue samples of invasive breast cancer from the pathology department of Qingdao Central Hospital in 2021 were screened. After HE staining, HE stained sections were irradiated with sunlight to simulate natural fading, and every 7 days was a fading cycle, and a total of 8 cycles were experienced. At the end of each cycle, the sections were digitally scanned to retain clear images, and the color changes of the sections during the fading process were recorded. The color transfer algorithm was applied to restore the color of the faded images; Adobe Lightroom Classic software presented the histogram of the image color distribution; UNet++ cell recognition segmentation model was used to identify the color restored images; Natural Image Quality Evaluator (NIQE), Information Entropy (Entropy), and Average Gradient (AG) were applied to evaluate the quality of the restored images.

**Results:**

The restored image color met the diagnostic needs of pathologists. Compared with the faded images, the NIQE value decreased (*P*<0.05), Entropy value increased (*P*<0.01), and AG value increased (*P*<0.01). The cell recognition rate of the restored image was significantly improved.

**Conclusion:**

The color transfer algorithm can effectively repair faded pathology images, restore the color contrast between nucleus and cytoplasm, improve the image quality, meet the diagnostic needs and improve the cell recognition rate of the deep learning model.

## Introduction

Hematoxylin-eosin staining (HE) is a routine staining method for pathological sections, which can better show the tissue structure and cell morphology and can be used to observe and describe the morphology of normal and diseased tissues. Pathology sections have two general uses: clinical pathology diagnosis and pathology teaching. H&E-stained slides carry pathological information about the patient and provide a reliable basis for diagnosing the disease and guiding subsequent treatment. It is estimated that there will be about 19.2 million new cases of cancer worldwide in 2020, an increase of 1.1 million from 18.1 million in 2018 [1]. The biological information contained in the pathological section is an important basis for the pathologist to make a diagnosis. It is roughly estimated that each tumor patient will produce 5 pathological slides during the diagnosis process, so at least 96 million pathological slides will be produced each year. In addition to the diagnostic role, pathological sections are also an important basis for differentiation [2–5]. Each section carries unique pathological information, however, if not preserved properly, the original HE-stained sections will fade or even completely fade over time, resulting in the loss of valuable pathological information. Therefore, it is essential to restore the pathological information in these faded sections.

The concept of color transfer was first proposed by Reinhard in 2001 [6]. Color transfer refers to the process of transferring the color of the target image to the original image to obtain a color-repaired image. Image enhancement algorithms have been widely used in image restoration tasks [7, 8]. The color transfer algorithm belongs to a means of image enhancement, which is an important way to change the expression of image information in image processing. This method is suitable for scenarios where both the reference image and the input image have a single-color distribution. When the color distribution in the image is rich, a comparison between the resulting image obtained by Reinhard’s algorithm and the original input image reveals that the color transfer does not achieve the purpose of changing the overall color balance of the image. Later Welsh proposed a remap grayscale image transfer algorithm based on the color transfer algorithm [9]. Grayscale images have only luminance information, so the algorithm achieves automatic colorization of grayscale images mainly by matching the luminance values of pixels. In this paper, we aim to explore a restoration method based on faded digital pathology images, which can avoid the tedious re-staining process, protect the tissue sections from damage by the re-staining process, and restore the red-blue contrast and original pathological features of the sections.

## Information and methods

### HE staining and light fading of pathological sections

Twenty fresh tissue samples of invasive breast cancer were collected in 2021, all from female patients aged≥50 years with a mean age of (54.63±4.36) years. The lesion tissues were fixed and hardened using the paraffin embedding method, cut into 4-μm slices using a microtome, and the wax slices were then picked up with forceps and laid flat on the surface of water at 40–45°C. The wax strips were left to spread naturally, and the slices were retrieved and dried before being fully automatically stained using a Roche Ventana HE600 staining machine. Sections with a score greater than 90 were included in the study according to the scoring criteria outlined in Table 1 [10].

**Table 1:** Routine paraffin-embedded-basic criteria for the quality of HE-stained sections.

number	Premium Standard	full score	Quality Defect Deduction
①	The tissue section is complete, and the number of sections of endoscopic bite and puncture specimens is complete	10	Slightly incomplete organization: minus 1~3 points; incomplete; minus 4~10 points; not reaching the required number of surfaces: minus 5 points
②	Thin slices (3~5μm), uniform thickness	10	Thick slices (overlapping cells), affecting the diagnosis: minus 6~10 points; uneven thickness: minus 3~5 points
③	Slice without knife marks and cracks	10	There are knife marks and fissures, but not affecting the diagnosis: minus 2 points; affecting the diagnosis: minus 5 points
④	Slices are flat, without wrinkles and folds	10	There are wrinkles or folds, but not affecting the diagnosis: minus 2 points; affecting the diagnosis: minus 5 points each
⑤	Slice free of contamination	10	Contaminants: minus 10 points
⑥	No air bubbles (between sections and slides/coverslips and sections and slides), and no glue spills around the coverslips	10	There are bubbles: minus 3 points; glue overflow: minus 3 points
⑦	good transparency	10	Poor transparency: minus 1~3 points; blurred organizational structure: minus 5~7 points
⑧	Clear contrast between nucleus and cytoplasm staining	10	The nucleus is grayish or too blue: minus 5 points; the contrast between red (cytoplasm) and blue (nucleus) is not clear: minus 5 points
⑨	The slices are not loose, and the mounting position is appropriate	10	Loose slices: minus 5 points; improperly mounted slices: minus 5 points
⑩	Neatly sliced, well-labeled, and clearly numbered	10	Untidy slices: minus 3 points; sticky labels: minus 3 points; unclear number: minus 4 points
total		100	

Note: Slice quality grading standards: ① Grade A slice: ≥ 90 points (excellent); ② Grade B slice: 75–89 points (good) ③ Grade C slice: 60–74 points (basic qualified); ④ Grade D slice: ≤ 59 points (unqualified)

**Fig 1:**
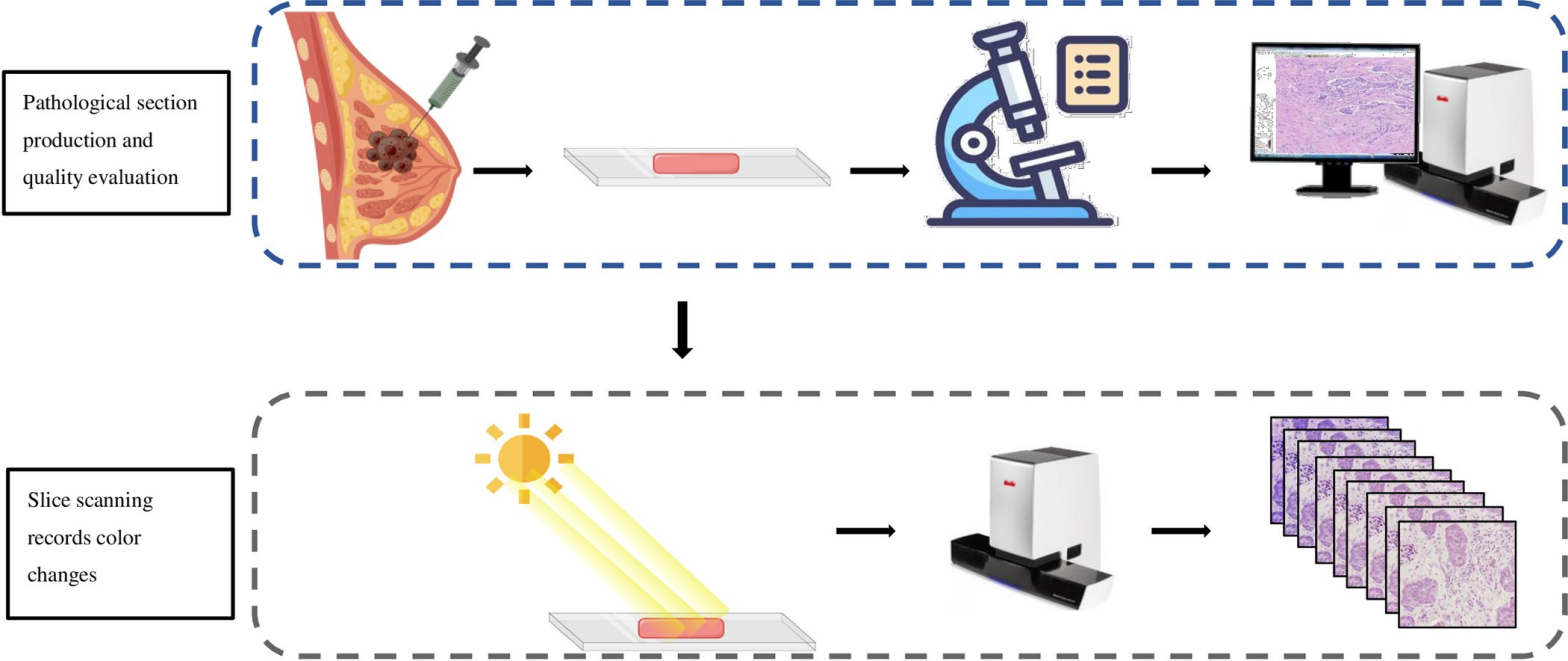
Slicing and digital image creation process. Sections with a score greater than 90 were included in the study. After HE staining, HE stained sections were irradiated with sunlight to simulate natural fading, and every 7 days was a fading cycle, and a total of 8 cycles were experienced. At the end of each cycle, the sections were digitally scanned to retain clear images, and the color changes of the sections during the fading process were recorded.

After HE staining, the sections were irradiated with sunlight to simulate natural fading. Each fading cycle lasted for 7 days, and a total of 8 cycles were completed. At the end of each cycle, the sections were digitally scanned to retain clear images, and the color changes of the sections during the fading process were recorded. Sunlight was chosen to discolor the slices because this method is simple, does not require opening the closed slices, avoids damage to the slices, and is more in line with the natural discoloration process of the slices. All slices are scanned using the Motic EasyScanner HD pathology section scanner, which is equipped with a fully automatic scanning platform: main camera: 2/3-inch CCD chip; resolution: 2448×2048; focus camera: CCD chip, resolution≥1360×1024; macro camera: CCD chip, resolution: 2048×1536. There are two digital image resolutions to choose from when scanning: 20X: 0.24um/pixel, and 40X: 0.12um/pixel, and we choose to scan the image at 0.12um/pixel resolution. We finally screened HE pathology images from 6 patients and selected 12 ROI regions from them for study, with a size of 672×672 pixels, and intercepted the corresponding ROI region at the same position of the faded image in each cycle to facilitate our color control, (Fig 1).

### Color transfer algorithm and cell recognition segmentation model

The color transfer algorithm is borrowed from Reinhard’s algorithm, which means that the transferred image acquires the colors target image while preserving the structure of source image.

The basic idea is to determine a linear transformation based on the statistical analysis of the colored image, so that the target image and the source image are in laβ having the same mean and variance in space. Assuming l, a, and b are the source images lαβ. The original data of the channel, L, A, and B are the transformed images obtained after transformation lαβ. The values of the channels, ml, ma, mb and ml’, ma’, mb’ are the mean values of the three color channels of the source and target images, respectively. nl, na, nb and nl’, na’, nb’ represent their standard deviations. The formulas for transformation are:

L=(nl'/nl)*(l–ml)+ml'

A=(na'/na)*(a–ma)+ma'

B=(nb'/nb)*(b–mb)+mb'

**Fig 2:**
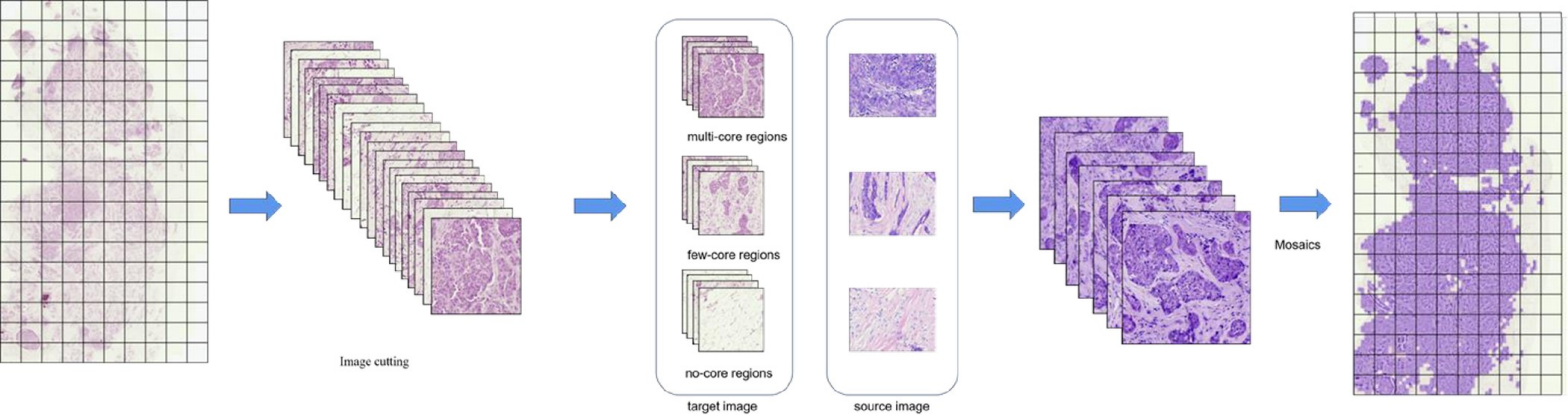
Color migration flowchart. Calculate the mean and standard deviation of two images, and determine a linear transformation based on the statistical analysis of the colored image, so that the target image and source image are in a linear transformation la13 having the same mean and variance in space.

We also use prior information to classify the image into multi-core regions, few-core regions and no-core regions. Furthermore, to enhance the contrast between the nucleus and the cytoplasm, the algorithm incorporates a contrast enhancement algorithm to improve the visual quality of the slice (Fig 2). UNet++ model was chosen to segment the cell and to verify whether the restored images can be used for intelligent diagnosis as well [11–13].

### Image evaluation

NIQE (Natural Image Quality Evaluator) [14], Entropy (Information Entropy) [15], and AG (Average Gradient) [16] were applied to evaluate the image quality before and after restoration. NIQE is a collection of "quality-aware" statistical features based on the natural scene statistics (NSS) model in the spatial domain, which can evaluate the image quality without a reference image. The smaller the NIQE value, the higher the image quality. AG can be used to measure the clarity of an image, and the higher the value, the clearer the image.

### Statistical treatment

SPSS 26.0 software was applied for statistical analysis. The measurement data were expressed as mean ± standard deviation or median quartiles, and the comparison of image quality before and after restoration was based on whether the difference conformed to a normal distribution using the t-test for two paired samples and the signed rank-sum test for paired data, respectively. *P* < 0.05 indicated that the difference was statistically significant.

## Results

### Faded images and RGB curves

**Fig 3:**
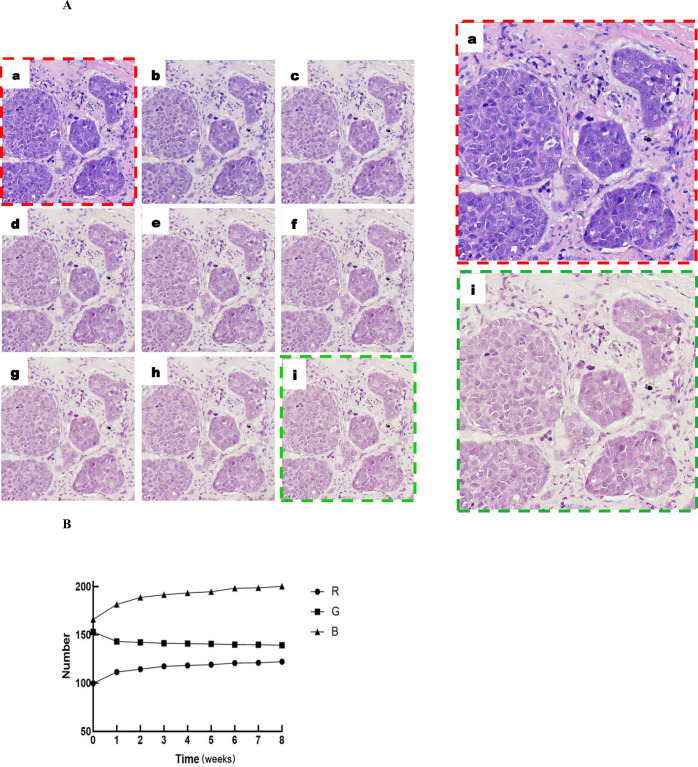
Digital images of pathology sections after 8 fading cycles. A: Color change of pathology sections after light treatment: (a) digital pathology images after fully automated staining by Roche Ventana HE600 staining machine. (b)-(i)digital scanned images at the end of each fading cycle. B: Folding graph of RGB value change of fading images.

Hematoxylin-eosin staining is both a standard stain for human histological examination and reveals cellular details. After staining the tissue with hematoxylin-eosin, the nucleus is blue, and the cytoplasm and extracellular matrix are pink. The images are brightly colored and contrasting before the start of the fading cycle, allowing the intrinsic structure of the cells to be observed. At the end of the first fading cycle, the cytoplasm and extracellular matrix fade significantly, and eosin is light-sensitive and easily fades in the case of closed sections. The color of the nucleus changed little and gradually turned pale pink with the growth of the fading cycles. The faded images make disease diagnosis difficult. (a) is the section image after automatic staining, (b)-(i) are the scanned images of sections after 1~8 fading cycles, and the fading curves are plotted based on the RGB values of the images (Fig 3).

### Image visual contrast and RGB histogram

**Fig 4:**
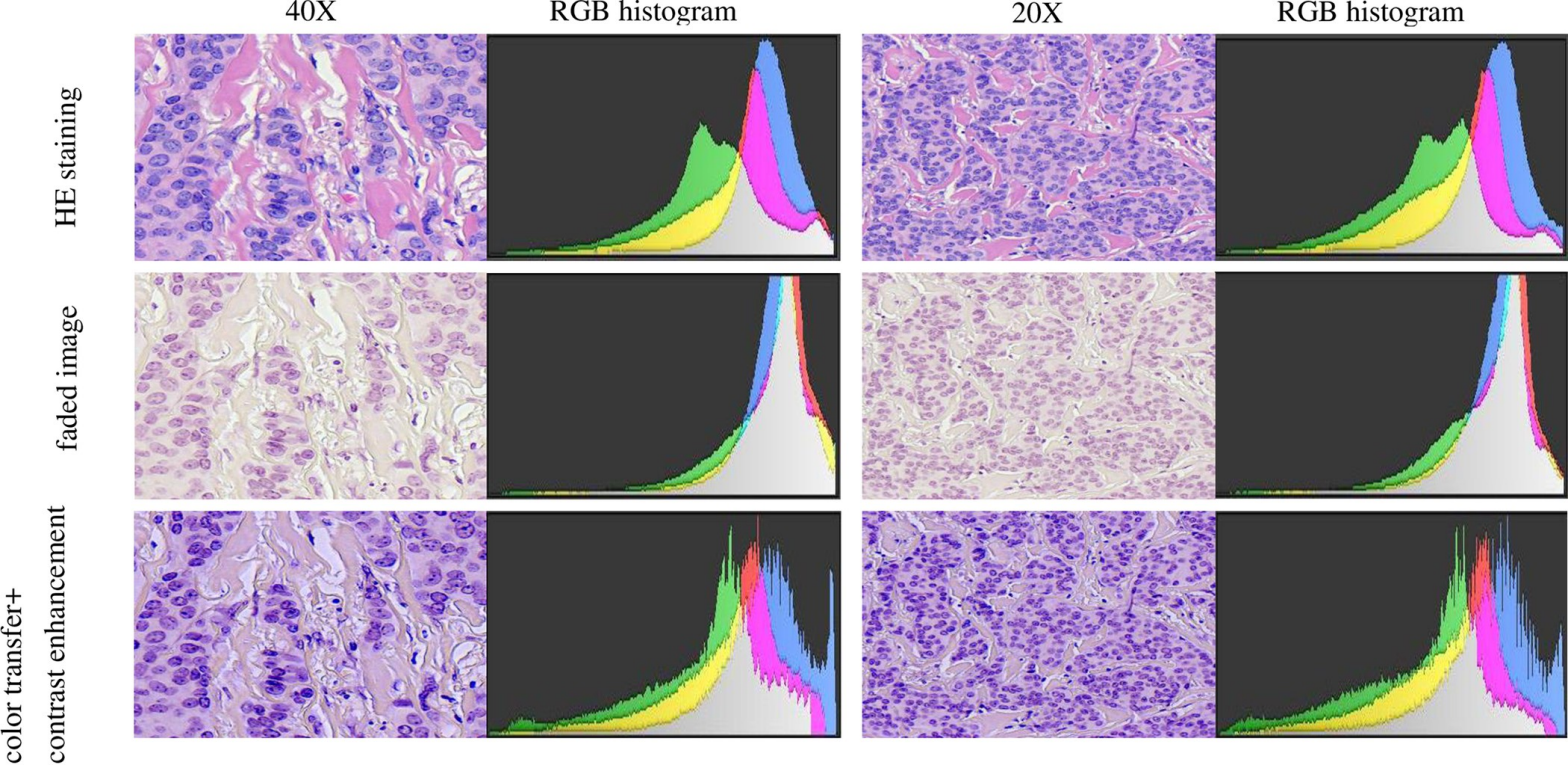
Histogram of HE, faded and restored pathology images and RGB color distribution.

The faded image was restored with color transfer and contrast enhancement algorithms, and the restored image restored the pathology image color to clearly observe the cell structure, and after two pathologists judged that the restored pathology image color and cell structure could meet the diagnostic needs. We also applied the RGB histogram to objectively and directly evaluate the image color information by displaying the R channel, G channel, and B channel information in the histogram, respectively. RGB histograms are red, green, and blue histograms overlapped together, where the overlapping parts will show the corresponding color. The part of the red histogram overlapped with the green histogram is yellow, the part of the blue histogram overlapped with the green histogram is cyan, the part of the red histogram overlapped with the blue histogram is magenta, and gray-green is the part of all three histograms overlapped together. In the histogram pre-faded images with bright color, the R, G, and B channels partially overlapped. After fading, the image color was single overall pink and lacked red and blue contrast, so the R, G, and B channels in the histogram were highly overlapped with gray color. The RGB histogram of the repaired image shows that the overlapping area of the three color channels is reduced, which is closer to the distribution structure of the histogram before the fading (Fig 4).

### Image quality evaluation

**Fig 5:**
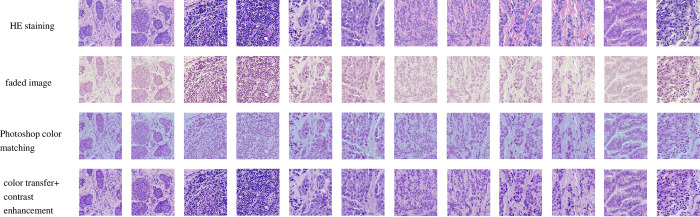
Color transfer algorithm and photoshop for color fading inpainting restoration results.

We first conducted a visual comparison of the repaired images. Compared to Photoshop, our method is more natural in color restoration and highlights the details of cell structure (Fig 5). The objective evaluation indicators of the image also show that color restoration significantly improves the quality of the image.

Compared with the faded image (B), the restored image (C) shows a significant decrease in NIQE and an increase in Entropy and AG, all of which have statistical differences. It is worth noting that there is no statistical difference in NIQE and Entropy between the restored image (C) and the HE-stained image (A). NIQE can evaluate the image quality of super-resolution images without a reference image, and a smaller value indicates better image quality. Entropy represents the rich information contained in the image. The larger the value, the more information the image contains and the better the image quality. The AG represents the sharpness of the image, and a larger value indicates a clearer image. The sharpness of the faded image is significantly reduced, while the restored image is enhanced by the contrast enhancement algorithm. Therefore, the clarity of the restored image is significantly improved, even exceeding that of the faded image. The color transfer and enhancement algorithm effectively restored the color and information of the faded image, and there is no significant difference in both NIQE and Entropy between the restored image and the HE-stained image (Table 2).

**Table 2:** Image quality comparison.

number	NIEQ		NIEQ		NIEQ		entrory		entrory		entrory		AG		AG		AG	
	A	B	B	C	A	C	A	B	B	C	A	C	A	B	B	C	A	C
1	17.75	18.31	18.31	16.05	17.75	16.05	7.22	6.55	6.55	7.31	7.22	7.31	12.56	10.17	10.17	15.62	12.56	15.62
2	19.35	18.92	18.92	16.73	19.35	16.73	7.35	6.70	6.70	7.46	7.35	7.46	12.95	10.44	10.44	16.17	12.95	16.17
3	20.77	20.05	20.05	18.80	20.77	18.80	7.80	7.47	7.47	7.47	7.80	7.47	17.46	14.92	14.92	15.96	17.46	15.96
4	16.92	18.71	18.71	17.05	16.92	17.05	7.86	7.60	7.60	7.46	7.86	7.46	18.02	17.30	17.30	18.37	18.02	18.37
5	23.68	28.25	28.25	22.61	23.68	22.61	7.43	6.84	6.84	7.38	7.43	7.38	10.21	8.20	8.20	10.66	10.21	10.66
6	25.13	27.96	27.96	27.17	25.13	27.17	7.35	6.68	6.68	7.37	7.35	7.37	8.33	7.24	7.24	9.52	8.33	9.52
7	15.83	19.63	19.63	17.08	15.83	17.08	7.31	6.60	6.60	7.43	7.31	7.43	15.71	12.44	12.44	20.00	15.71	20.00
8	16.19	14.98	14.98	13.12	16.19	13.12	7.32	6.64	6.64	7.36	7.32	7.36	16.06	13.32	13.32	21.29	16.06	21.29
9	23.52	22.62	22.62	19.59	23.52	19.59	7.36	6.66	6.66	7.42	7.36	7.42	11.74	9.12	9.12	14.12	11.74	14.12
10	22.63	24.75	24.75	24.03	22.63	24.03	7.22	6.55	6.55	7.32	7.22	7.32	10.33	8.69	8.69	13.55	10.33	13.55
11	20.30	21.28	21.28	18.66	20.30	18.66	7.35	6.51	6.51	7.43	7.35	7.43	14.16	13.50	13.50	25.19	14.16	25.19
12	22.98	24.12	24.12	21.86	22.98	21.86	7.50	7.10	7.10	7.43	7.50	7.43	8.76	8.61	8.61	10.11	8.76	10.11
*t/Z*	-2.228		-3.061		1.893		-3.064		-2.851		-0.628		6.498		-5.007		-3.113	
*P*	0.048		0.002		0.085		0.002		0.004		0.530		<0.001		<0.001		0.010	

Note: A: HE staining; B: faded image; C: color transfer+contrast enhancement

### Cell recognition results with an artificial intelligence algorithm

In our breast cancer pathological image classification task, we utilized ResNet101 as the backbone architecture and processed the images at their original size of 672 × 672 pixels. Given the unique characteristics of breast cancer pathology images, we developed a Discrimination Enhancement Module (DEM) that enhances the margin between different classes in the feature space. We achieved this by applying channel attention and spatial attention techniques to highlight the features of the foreground object in all of the channels. Specifically, the channel attention mechanism selectively emphasizes informative features in each channel by applying a global pooling operation followed by a fully connected layer and a sigmoid activation. Meanwhile, the spatial attention mechanism identifies the most relevant regions in the image by performing convolutional operations across the spatial dimensions, and then applying a softmax activation to obtain a spatial attention map. By combining these two techniques, we were able to improve the distinction among different classes and achieve higher classification performance in our breast cancer pathological image classification task.

**Fig 6:**
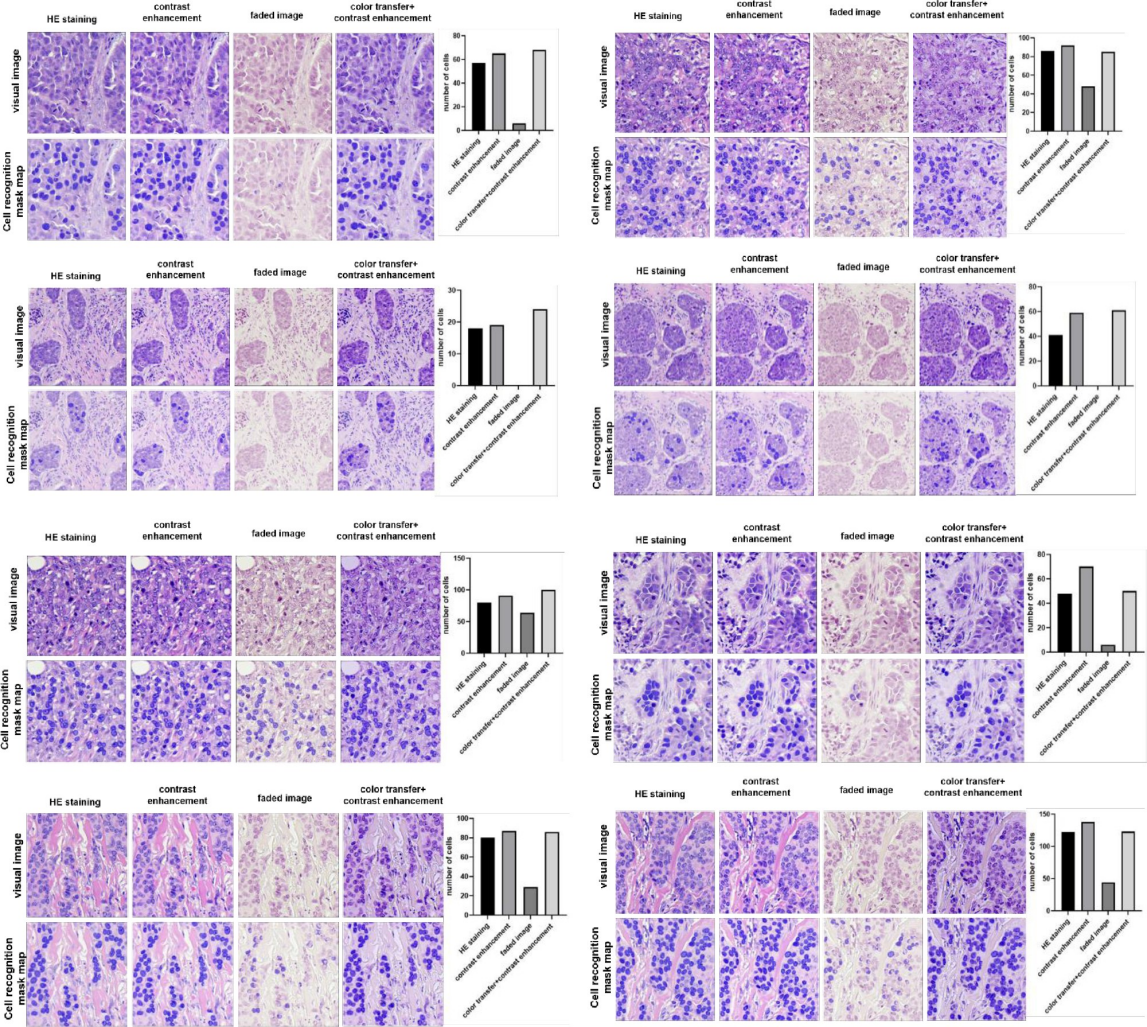
UNet++ model cell identification results.

We selected 8 regions rich in tumor cells for counting, and applying the enhancement algorithm to the pre-faded images alone can improve cell recognition to some extent. The faded images were very poor in cell recognition because of the loss of color contrast, and there was even no way to recognize the cells in some of the images. After applying the color transfer and contrast enhancement algorithm, the image quality and visual effect are effectively restored. The cell recognition rate of the restored image is basically the same as the cell recognition rate before fading. The restored images can meet the requirements of both visual and deep learning, and meet the needs of multiple uses of images (Fig 6).

### Discussion

Survival of tumor patients is significantly longer with the improvement of medical treatment. However, some patients may develop new tumors in other parts of the body during their survival period. To clarify whether the newly discovered tumor is a primary tumor in that organ or whether the previous tumor has metastasized, it is necessary to compare the tissue section of the newly discovered tumor with the pathological section of the previous one. However, due to the light and humidity of the storage environment, HE staining of pathology sections faded. Faded pathology sections pose difficulties in diagnostic and differential diagnosis work. Previously, the main method to solve the problem of discoloration of pathology sections in clinical work was to stain again, and the main process included: first, the discolored sections were soaked in xylene for 72h and then the coverslips were removed, and then it was necessary to continue to soak in xylene for 24h to remove the residual gum on the tissue surface, then soak in anhydrous ethanol for 2min, soak in 95% ethanol for 1min, rinse with water, then soak in citric acid solution for 15min, and finally rinse with water and then stain the tissue slides with hematoxylin-eosin [17]. Pathology section re-staining technique can recolor the tissue, but there is a risk of dislodging the tissue section during the removal of the coverslip and the process takes a long time, in addition to the molecular structure of the discolored tissue changes resulting in uneven tissue coloration affecting the diagnosis. Even if the faded sections are re-stained, permanent preservation is still not possible. Applying color transfer algorithms to repair pathology sections avoids damage to section parenchyma, reduces the use of chemical reagents, and reduces the risk and danger of occupational exposure to chemical reagents for pathologists. Applying color transfer algorithm to repair a faded digital slice only takes 30 mins, The restored digital images no longer face fading problems and can be stored permanently, enabling the accumulation of pathological images big data, increasing the number of rare pathology images and enriching the variety of pathology teaching images.

Digital pathological images are different from ordinary images. On the one hand, each digital pathological image contains billions of pixels, which is significantly larger than ordinary images. On the other hand, digital pathology images also contain cell level fine structures. Existing image processing software cannot process the entire digital pathology images, and the color normalization algorithm proposed by Zarella and Janowczyk et al. [18, 19]. has the problem of over dependence on the original image and information loss of the processed image. Therefore, the difficulty of processing huge images and restoring the image color while also effectively preserving the fine structure of the image is the difficulty and focus of digital pathology image restoration. To solve this problem, we restored the images of faded slices by dividing the whole image into many patches, and then dividing the patches into multi-core regions, few-core regions and no-core regions according to the number of nuclei contained in the image. The RGB value characteristics of these three areas are counted, and template matching is performed based on the RGB value information, and then the faded image is restored. This solves the problem of difficult computer processing of overly large digital pathology images and avoids the image distortion problems associated with using a single template. Our image restoration algorithm effectively restores the color of pathology images, while also applying contrast enhancement algorithms to significantly improve image quality.

Digital pathological images are widely used. Apart from artificial diagnosis and pathological teaching, they are most widely used in the field of artificial intelligence diagnosis. The realization of artificial intelligence diagnosis cannot be separated from the recognition and segmentation of cells by network models. At present, the network models with good segmentation effects for pathological images mainly include VGG, ResNet, GoogLeNet, DenseNet and UNet++ [20–24]. The excellent results shown by the network model are inseparable from the good image quality. In order to verify whether the quality of our restoration image meets the requirements of the deep learning network model, we choose the UNet++ model to recognize the pathology images cells for segmentation, and the results show that we repair pathological images with excellent cell recognition results. We also note that applying the contrast enhancement algorithm alone on top of the original image improves the cell recognition rate, which suggests the need to pre-process the image before the image study, as this can enhance our experimental results. Because of the differences between pathology image datasets, some scholars normalized the images to reduce the differences between them. Color normalization of digital pathology images can effectively improve the visual quality of the images but they only adjusted for color [25–28]. Changing the color while increasing the contrast to highlight structural details can enhance the model results more effectively. Applying multi-stage detection methods and using multiple thresholds to extract texture information at different levels for clustering pathological images to improve cell detection rate [29, 30].

## Conclusion

Limitations of this study: only breast cancer pathology sections were repaired, and this method can be extended to other tumor sections. In summary, the application of a color transfer algorithm for color recovery of faded sections can restore the red-blue contrast of the sections, which meets the requirements of medical pathology images and facilitates retrospective evaluation by pathologists. We should actively promote the digitization process of pathology slides and realize digital permanent preservation.

## Supporting information

S1 Fig Fading image dataset. (ZIP)

S1 File Code for color transfer. (DOCX)
