# Supplementary figures and images for: Color restoration based on digital pathology image
Source: PLoS One. 2023 Jun 28;18(6):e0287704. doi: 10.1371/journal.pone.0287704 (PMC10306179; doi:10.1371/journal.pone.0287704)

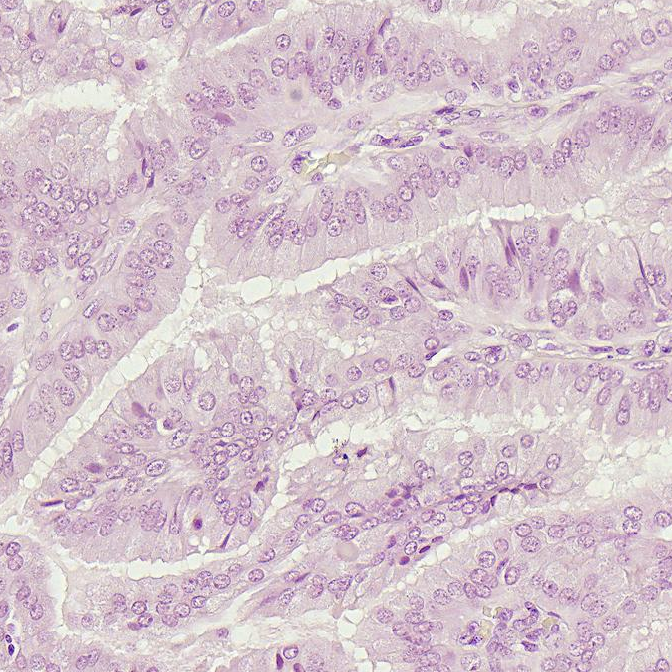

Supplement: S1 Fig — (ZIP) [file pone.0287704.s001.zip › S1 Fading Image Dataset/20-1-9_0_0.png]

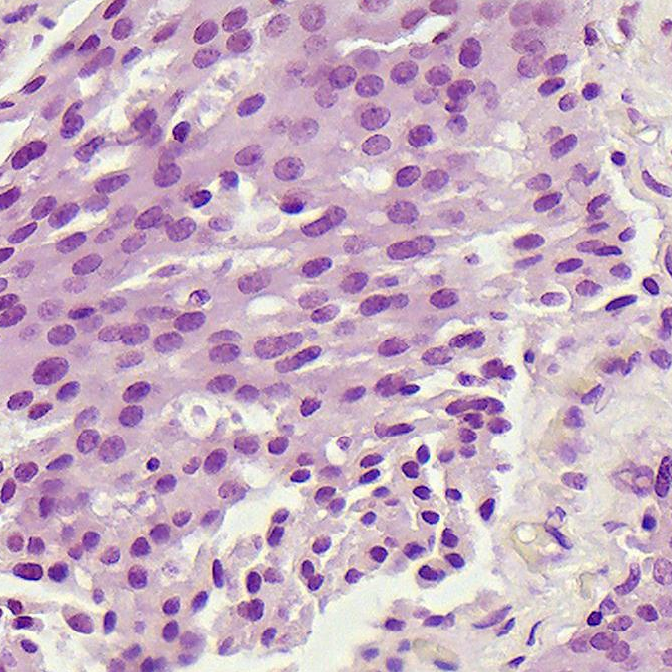

Supplement: S1 Fig — (ZIP) [file pone.0287704.s001.zip › S1 Fading Image Dataset/20-2-9_0_0.png]

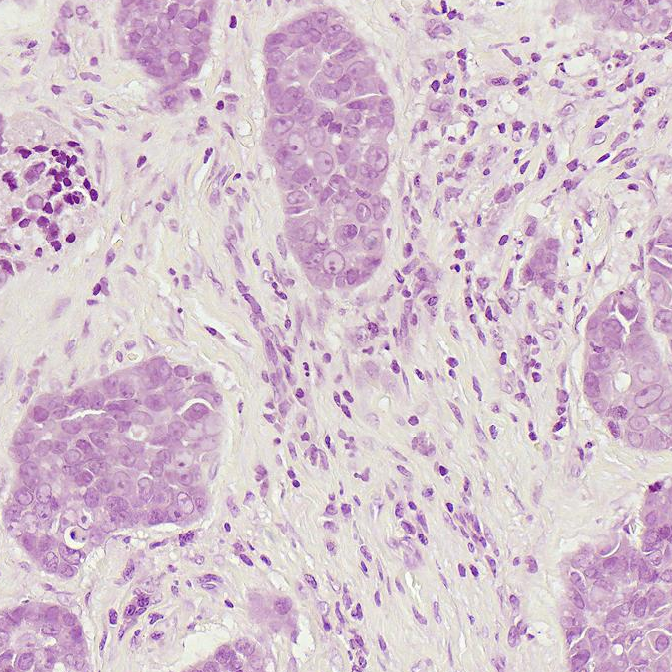

Supplement: S1 Fig — (ZIP) [file pone.0287704.s001.zip › S1 Fading Image Dataset/5-1-9_0_0.png]

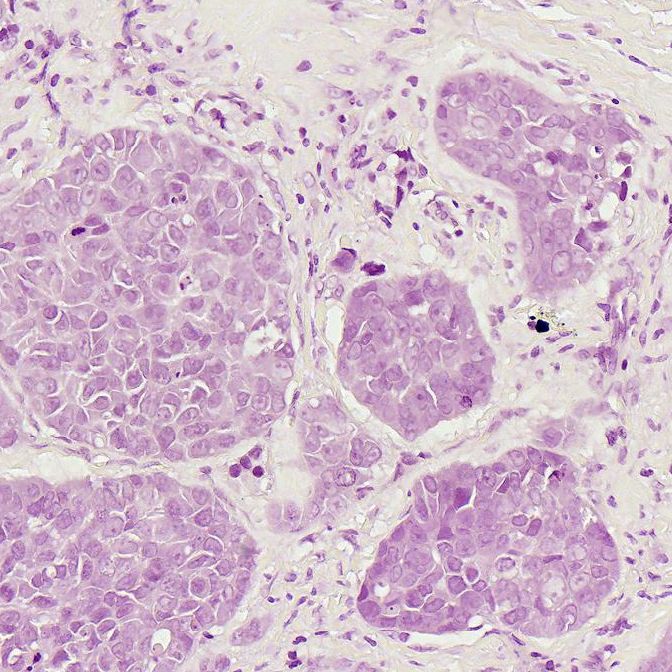

Supplement: S1 Fig — (ZIP) [file pone.0287704.s001.zip › S1 Fading Image Dataset/5-1-9_0_1.png]

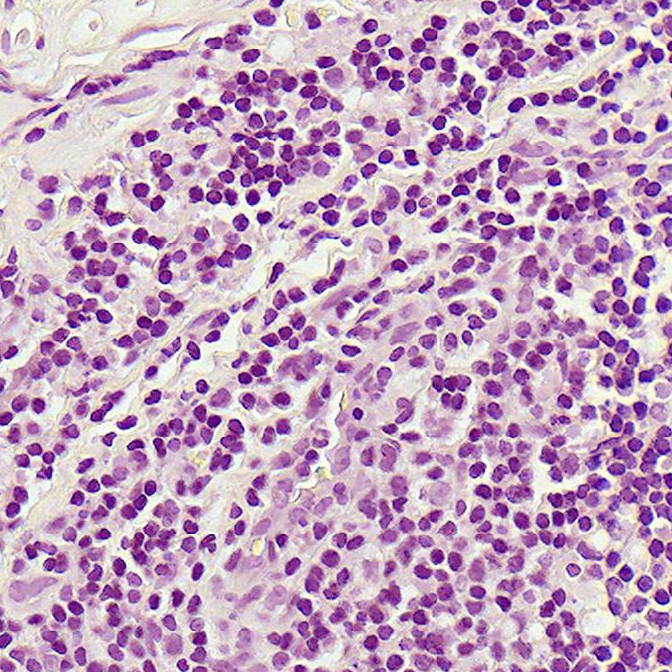

Supplement: S1 Fig — (ZIP) [file pone.0287704.s001.zip › S1 Fading Image Dataset/5-2-9_0_0.png]

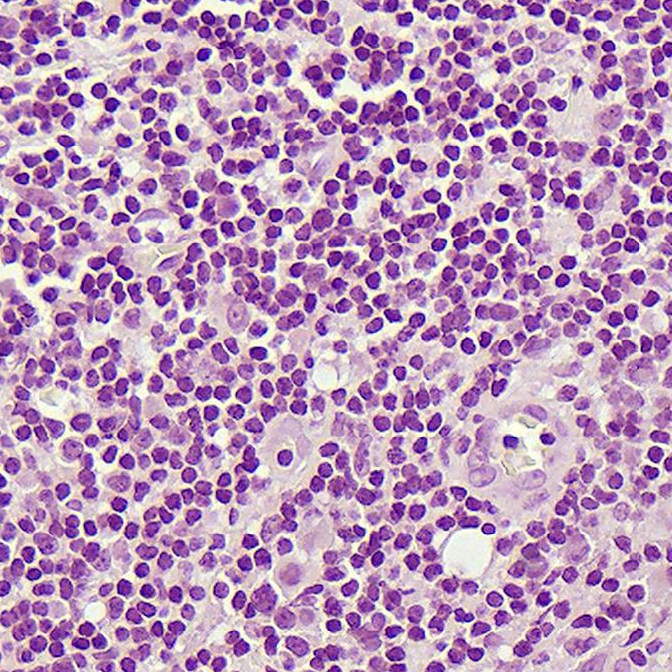

Supplement: S1 Fig — (ZIP) [file pone.0287704.s001.zip › S1 Fading Image Dataset/5-2-9_0_1.png]

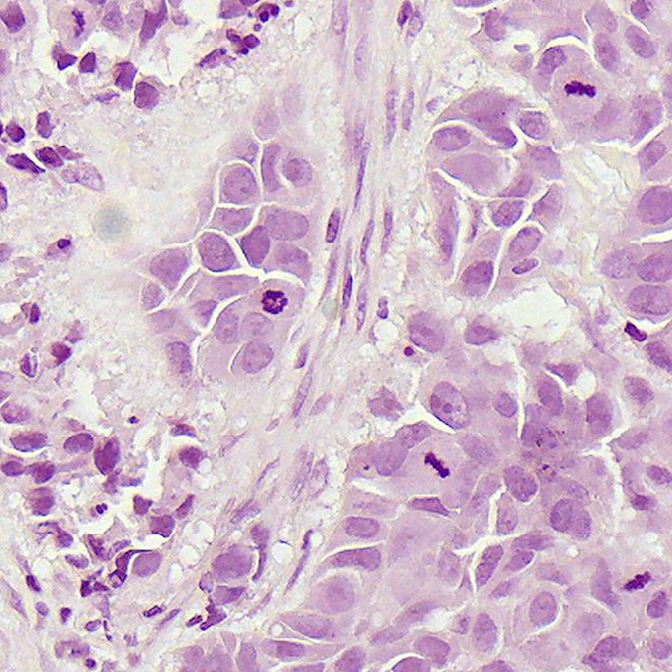

Supplement: S1 Fig — (ZIP) [file pone.0287704.s001.zip › S1 Fading Image Dataset/6-1-9_0_0.png]

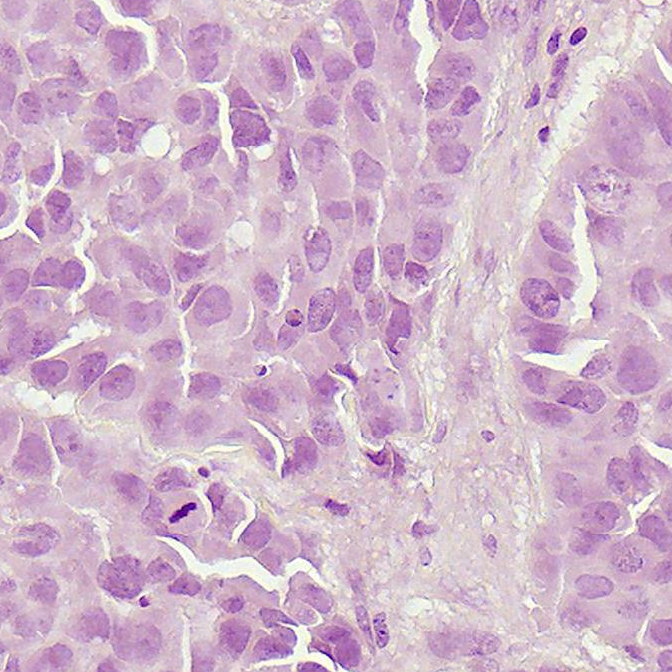

Supplement: S1 Fig — (ZIP) [file pone.0287704.s001.zip › S1 Fading Image Dataset/6-1-9_0_1.png]

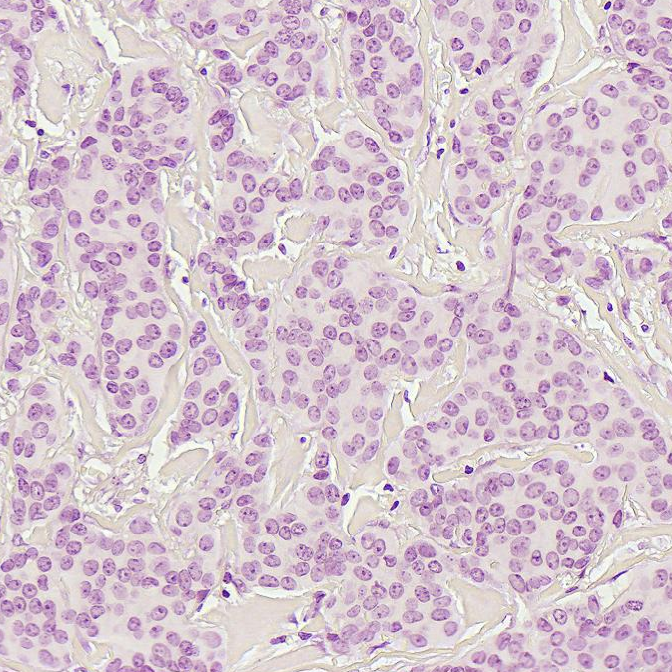

Supplement: S1 Fig — (ZIP) [file pone.0287704.s001.zip › S1 Fading Image Dataset/8-1-9_0_0.png]

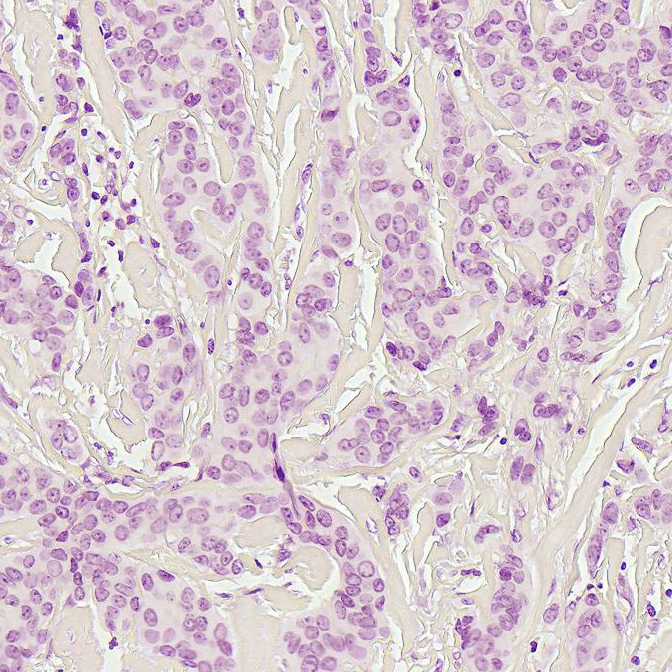

Supplement: S1 Fig — (ZIP) [file pone.0287704.s001.zip › S1 Fading Image Dataset/8-1-9_0_1.png]

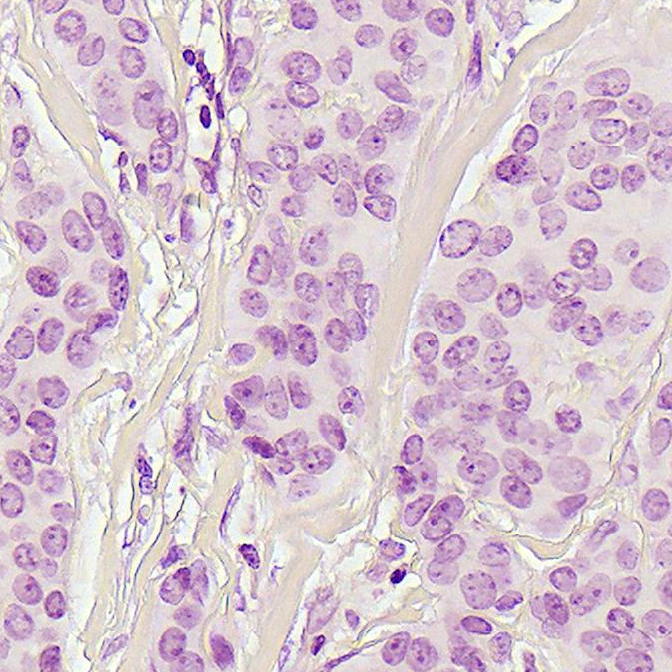

Supplement: S1 Fig — (ZIP) [file pone.0287704.s001.zip › S1 Fading Image Dataset/8-2-9_0_0.png]

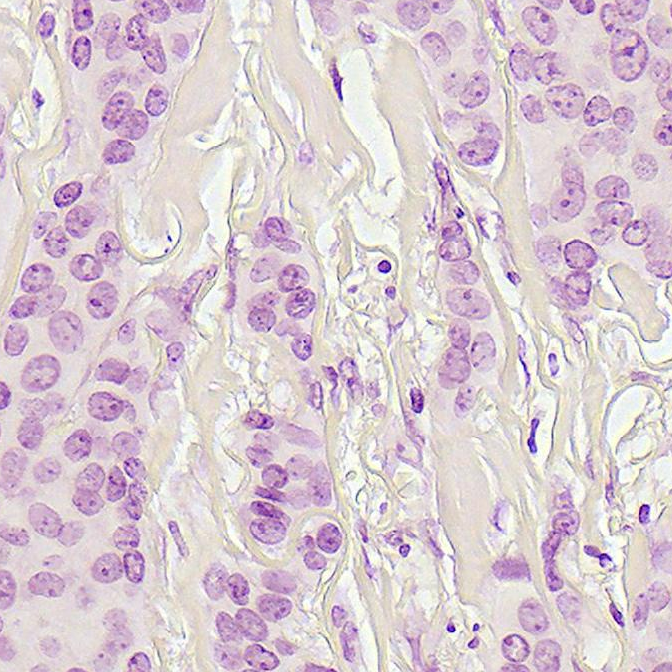

Supplement: S1 Fig — (ZIP) [file pone.0287704.s001.zip › S1 Fading Image Dataset/8-2-9_0_1.png]
