# Supplementary material for: Color restoration based on digital pathology image
Source: PLoS One. 2023 Jun 28;18(6):e0287704. doi: 10.1371/journal.pone.0287704 (PMC10306179; doi:10.1371/journal.pone.0287704)
Supplement: S1 File — (DOCX) [file pone.0287704.s002.docx]

import numpy as np
import cv2


image = cv2.imread("C:/Users/LiFei/Desktop/test/000raw-890/521.png")
# cv2.imshow("src1",image)
image = cv2.cvtColor(image, cv2.COLOR_BGR2LAB)
original = cv2.imread("C:/Users/LiFei/Desktop/762.png")
# cv2.imshow("original",original)
original = cv2.cvtColor(original, cv2.COLOR_BGR2LAB)

# 得到均值和标准差
def getavgstd(image):
 avg = []
 std = []
 image_avg_l = np.mean(image[:, :, 0])
 image_std_l = np.std(image[:, :, 0])
 image_avg_a = np.mean(image[:, :, 1])
 image_std_a = np.std(image[:, :, 1])
 image_avg_b = np.mean(image[:, :, 2])
 image_std_b = np.std(image[:, :, 2])
 avg.append(image_avg_l)
 avg.append(image_avg_a)
 avg.append(image_avg_b)
 std.append(image_std_l)
 std.append(image_std_a)
 std.append(image_std_b)
 return (avg, std)


image_avg, image_std = getavgstd(image)
original_avg, original_std = getavgstd(original)

height, width, channel = image.shape
for i in range(0, height):
 for j in range(0, width):
 for k in range(0, channel):
 t = image[i, j, k]
 t = (t - image_avg[k]) * (original_std[k] / image_std[k]) + original_avg[k]
 t = 0 if t < 0 else t
 t = 255 if t > 255 else t
 image[i, j, k] = t
image = cv2.cvtColor(image, cv2.COLOR_LAB2BGR)
# cv2.imshow("dst",image)
cv2.imwrite("C:/Users/LiFei/Desktop/521to.png", image)
cv2.waitKey(0)
